# *Anaplasma phagocytophilum* strains from voles and shrews exhibit specific *ankA* gene sequences

**DOI:** 10.1186/1746-6148-9-235

**Published:** 2013-11-28

**Authors:** Juliana Majazki, Nicole Wüppenhorst, Kathrin Hartelt, Richard Birtles, Friederike D von Loewenich

**Affiliations:** 1 Institute of Medical Microbiology and Hygiene, University of Freiburg, Hermann-Herder-Strasse 11, Freiburg D-79104, Germany; 2 Baden-Wuerttemberg State Health Office, District Government Stuttgart, Nordbahnhofstrasse 135, Stuttgart D-70191, Germany; 3 School of Environment and Life Sciences, University of Salford, The Crescent, Salford M5 4WT, UK

**Keywords:** *Anaplasma phagocytophilum*, Voles, Shrews, Genotyping, *ankA* gene, Recombination

## Abstract

**Background:**

*Anaplasma phagocytophilum* is a Gram-negative bacterium that replicates obligate intracellularly in neutrophils. It is transmitted by *Ixodes* spp. ticks and causes acute febrile disease in humans, dogs, horses, cats, and livestock. Because *A. phagocytophilum* is not transmitted transovarially in *Ixodes* spp., it is thought to depend on reservoir hosts to complete its life cycle. In Europe, *A. phagocytophilum* was detected in roe deer, red deer, wild boars, and small mammals. In contrast to roe deer, red deer and wild boars have been considered as reservoir hosts for granulocytic anaplasmosis in humans, dogs, and horses according to *groESL*- and *ankA*-based genotyping. *A. phagocytophilum* variants infecting small mammals in Europe have not been characterized extensively to date.

**Results:**

We amplified the total *ankA* open reading frames of 27 strains from voles and shrews. The analysis revealed that they harboured *A. phagocytophilum* strains that belonged to a distinct newly described *ankA* gene cluster. Further, we provide evidence that the heterogeneity of *ankA* gene sequences might have arisen via recombination.

**Conclusions:**

Based on *ankA*-based genotyping voles and shrews are unlikely reservoir hosts for granulocytic anaplasmosis in humans, dogs, horses, and livestock in Europe.

## Background

*Anaplasma phagocytophilum* is a Gram-negative bacterium that replicates obligate intracellularly in neutrophils [1]. It is tick-transmitted and causes acute febrile disease in humans [2], in companion animals such as dogs [3], horses [4], and cats [5] as well as in livestock such as sheep and cattle [6,7]. The main vector of *A. phagocytophilum* in Europe is *Ixodes ricinus*, whereas it is primarily transmitted by *I. scapularis* and *I. pacificus* in North America and by *I. persulcatus* in Asia [2].

Evidence exists that the naturally circulating *A. phagocytophilum* strains show a considerable degree of host adaptation, because they are not equally infectious for different animal species [3,7-9]. The molecular characterization using major surface protein 2 (*msp2*) pseudogene sequences [10] as well as the *ankA* gene [11] has shown that strains originating from humans, dogs, and horses are homologous. Furthermore, horses and dogs are susceptible to infection with human *A. phagocytophilum* isolates [12-14].

At least in *Ixodes* spp. ticks *A. phagocytophilum* is not transmitted transovarially [15]. Therefore, it is thought to depend on reservoir hosts to complete its life cycle. In North America, based on molecular characterization and experimental infections small mammals such as white-footed mice [16,17], chipmunks [18,19], and squirrels [19] were reported as probable reservoirs for granulocytic anaplasmosis in humans, horses, and dogs. In contrast, the impact of white-tailed deer and woodrats was questioned [18,20,21]. In Europe, *A. phagocytophilum* was detected amongst others in roe deer [22,23], red deer [23], wild boars [24], hedgehogs [25], and other small mammals [26].

The 16S rRNA gene has been used most often for strain characterization. However, it was shown that it is not informative enough to delineate distinct *A. phagocytophilum* genotypes [11,27-29]. Based on *groESL* and *ankA* gene sequences red deer [11,30] and wild boar [31,32] were considered as reservoir hosts for granulocytic anaplasmosis in humans, dogs, and horses. In contrast, roe deer harboured *A. phagocytophilum* strains which mostly belonged to clearly separated *groESL*[30] and *ankA*[11] gene clusters.

Apart from using the 16S rRNA gene the *A. phagocytophilum* variants infecting small mammals in Europe have not been typed extensively to date. We therefore amplified the total *ankA* open reading frame (ORF) of 27 strains from voles and shrews captured in Germany as well as the UK and compared them to 221 *ankA* sequences determined earlier [11,27]. We here show that they harboured *A. phagocytophilum* strains that belonged to a distinct newly described *ankA* gene cluster. Therefore, voles and shrews are unlikely reservoir hosts for granulocytic anaplasmosis in humans, dogs, horses, and livestock in Europe.

## Methods

### Samples

27 *A. phagocytophilum* positive DNA samples from voles and shrews were investigated. 22 had been prepared earlier from the lung of voles captured in Germany [33]. Five had been purified from the blood of two voles [34] and three shrews [35] from the United Kingdom. The 16S rRNA and *ankA* gene sequences obtained here were compared to 221 sequences from humans, a great variety of animals, and *I. ricinus* ticks from previous studies [11,27]. Furthermore, seven additional samples from three humans, one dog, one horse, one cow, and one sheep were included. Table 1 shows host species and geographic origin of the samples.

**Table 1:** **Host species and geographic origin of *A. phagocytophilum *** **positive samples (n =34)**

**Sample**	**Origin**	**Sample**	**Origin**
*Myodes glareolus*		*Microtus arvalis*	
2/99	Germany	79/99	Germany
23/99	Germany	151/99	Germany
42/99	Germany	220/99	Germany
92/99	Germany	*Sorex araneus*	
106/99	Germany	S1	UK
129/99	Germany	S2	UK
159/99	Germany	S3	UK
240/00	Germany	*Homo sapiens*	
241/00	Germany	Human 96HE27	USA
252/00	Germany	Human 98HE4	USA
278/00	Germany	Human HGE-1^*^	USA
289/00	Germany	*Canis lupus familiaris*	
331/00	Germany	Dog Martin^**^	USA
338/00	Germany	*Equus caballus*	
354/00	Germany	Horse 32 FR	Switzerland
362/00	Germany	*Bos taurus*	
414/00	Germany	Cow A262	Germany
426/00	Germany	*Ovis aries*	
523/00	Germany	sheep F1480	Germany
*Microtus agrestis*			
F1	UK		
F6	UK		

^*^[36], ^**^[37].

### PCR analyses and sequencing

1 to 2 μl of DNA were used as template in a 50 μl reaction mixture containing 50 mM KCl, 20 mM Tris–HCl (pH 8.4), 2 mM MgCl_2_, 0.2 mM desoxynucleoside triphosphates, 0.4 μM of each primer, and 0.2 μl (1U) of *Taq* DNA Polymerase (Invitrogen, Karlsruhe, Germany). PCRs were performed using the GeneAmp PCR System 9700 (Applied Biosystems, Darmstadt, Germany) under the following conditions: initial denaturation at 94°C for 3 min, 40 cycles consisting of denaturation at 94°C for 30 s, annealing at the predicted melting temperature of the primers minus 4°C for 30 s, extension at 72°C for 30 s per amplification of 500 bp, and a final extension at 72°C for 10 min. Nested PCR amplification and sequencing of the *A. phagocytophilum* 16S rRNA gene [27,38] and of the *ankA* gene clusters I [39] and IV [11] were performed as described previously. Nested PCR amplification and sequencing of the *ankA* gene cluster V was achieved as shown in Additional file 1: Table S1. The sequence of the complete ORF was obtained by assembling the sequences of the six nested PCR products. Nucleotide sequences of primers (Metabion, Martinsried, Germany) are summarized in Additional file 2: Table S2. Nested PCR products were directly sequenced bidirectionally using a 3130 Genetic Analyzer (Applied Biosystems) and the BigDye Terminator v3.1 Cycle Sequencing Kit (Applied Biosystems).

### Data analysis

Sequences were edited and assembled with the SeqMan program of the DNASTAR package (Lasergene, Madison, WI). For phylogenetic analysis of the 16S rRNA or *ankA* gene sequences the program MEGA 5.1 [40] was used. Sequences were aligned by ClustalW applying the IUB matrix (16S rRNA gene) or codon-aligned applying the PAM (Dayhoff) matrix. Tree construction was achieved by the neighbor-joining method with the complete deletion option using the Jukes-Cantor matrix for nucleotide sequences and the PAM (Dayhoff) matrix for protein sequences, respectively. Bootstrap analysis was conducted with 1,000 replicates. Average distances within and net average distances between *ankA* gene clusters were computed using the same parameters as for tree construction. Protein sequences were analyzed for Pfam domain matches (http://pfam.sanger.ac.uk/) and for tyrosine kinase group phosphorylation sites (http://scansite.mit.edu/). Nucleotide consensus sequences were calculated for each *ankA* gene cluster with consensus maker v2.0.0 using the most common character and breaking ties with IUPAC characters (http://www.hiv.lanl.gov/content/sequence/HIV/HIVTools.html). The consensus sequences were codon-aligned by ClustalW applying the PAM (Dayhoff) matrix. The alignment was analyzed for recombination by Recco [41] with the Hamming mutation cost matrix and gap extension costs of 0.2. Events with seq *p*-values of < 0.5 and savings ≥ 5 were regarded as significant.

### Accession numbers

GenBank nucleotide accession numbers of 16S rRNA and *ankA* gene sequences are shown in Table 2.

**Table 2:** GenBank nucleotide accession numbers

**Sample**	**16S rRNA**	***ankA***	**Sample**	**16S rRNA**	***ankA***
**Voles**			**Voles**		
2/99	KC740418	KC740451	426/00	KC740435	KC740468
23/99	KC740419	KC740452	523/00	KC740436	KC740469
42/99	KC740420	KC740453	F1	KC740437	KC740470
79/99	KC740439	KC740472	F6	KC740438	KC740471
92/99	KC740421	KC740454	**Shrews**		
106/99	KC740422	KC740455	S1	KC740442	KC740475
129/99	KC740423	KC740456	S2	KC740443	KC740476
151/99	KC740440	KC740473	S3	KC740444	KC740477
159/99	KC740424	KC740457	**Humans**		
220/99	KC740441	KC740474	Human 96HE27	KC740446	KC740478
240/00	KC740425	KC740458	Human 98HE4	KC740447	KC740479
241/00	KC740426	KC740459	Human HGE-1	KC740445	KC740480
252/00	KC740427	KC740460	**Dog**		
278/00	KC740428	KC740461	Dog Martin	KC740448	KC740481
289/00	KC740429	KC740462	**Horse**		
331/00	KC740430	KC740463	Horse 32 FR	JN247407	JN247406
338/00	KC740431	KC740464	**Cow**		
354/00	KC740432	KC740465	Cow A262	KC740449	KC740482
362/00	KC740433	KC740466	**Sheep**		
414/00	KC740434	KC740467	Sheep F1480	KC740450	KC740483

## Results

### 16S rRNA gene sequences

Seven of the 16S rRNA gene sequences from voles contained ambiguous nucleotides, indicating multiple infections with several 16S rRNA genotypes, a phenomenon that was observed already earlier in animal and tick samples [11,27]. 14 of the 27 small mammals (11 voles and three shrews) harboured an *A. phagocytophilum* variant identical to [GenBank: M73220]. This genotype is widespread mainly in ruminants, but was also detected in voles and shrews [34,35,42]. Two 16S rRNA gene sequences were identical to [GenBank: AY082656] that was found in voles in the United Kingdom [43], whereas two matched [GenBank: GU236577] originating from red deer in Germany [11]. Additionally, one vole was infected with an *A. phagocytophilum* variant identical to [GenBank: AY281785] and one with a new variant, respectively.

### *ankA* sequences

Due to the pronounced dissimilarity of the *ankA* gene from voles and shrews to the known *ankA* gene clusters I, II, III, and IV described earlier [11,27], a new set of primers had to be developed for amplification and sequencing of the complete ORF (Additional file 1: Table S1). Despite the *ankA* sequence from one sheep that belonged to cluster IV, all other six samples from humans and animals analysed during this study were part of cluster I. The obtained *ankA* gene sequences from voles and shrews were 99.8% identical to each other at the nucleotide level and 99.6% similar at the protein level. The comparison to 221 sequences (12 from humans, 43 from dogs, 10 from horses, two from cats, 53 from sheep, four from cattle, 47 from roe deer, 12 from red deer, 15 from European bison, 23 from *I. ricinus* ticks) described earlier [11,27] indicated that the 27 samples from voles and shrews belonged to a new *ankA* gene cluster V and revealed nucleotide identities of 59.6% to 68.1% and amino acid similarities of 36.6% to 51.6% to the known clusters (Table 3). The sequences of *ankA* gene cluster V showed the lowest identities and similarities to all other *ankA* gene clusters indicating that they were most distantly related. The sequences most closely related to *ankA* gene cluster V were those from *ankA* gene cluster IV. However, their identity at the nucleotide level was limited to 68.1% and their similarity at the amino acid level to 51.6% (Table 3).

**Table 3:** Net average identities* and similarities** between the different *ankA * **gene clusters in percent**

	**Cluster I**	**Cluster II**	**Cluster III**	**Cluster IV**	**Cluster V**
**Cluster I**		85.4	74.6	69.6	61.3
**Cluster II**	*78.4*		83.3	73.5	63.0
**Cluster III**	*60.5*	*71.2*		65.7	59.6
**Cluster IV**	*59.8*	*63.3*	*48.3*		68.1
**Cluster V**	*45.0*	*44.7*	*36.6*	*51.6*	

*At the nucleotide level (roman), **at the protein level (italics).

A search against the Pfam domain database demonstrated that all AnkA sequences from voles and shrews contained ankyrin repeats. Furthermore, multiple tyrosine phosphorylation sites were predicted by Scansite (http://scansite.mit.edu/) at their C-terminal end, one of them displaying a classical EPIYA motif [44]. As described for AnkA clusters I and IV [11], the abundant tyrosine phosphorylation sites seemed to be arisen by duplication of direct repeats (Additional file 3: Figure S1).

### Phylogenetic analysis

**Figure 1:**
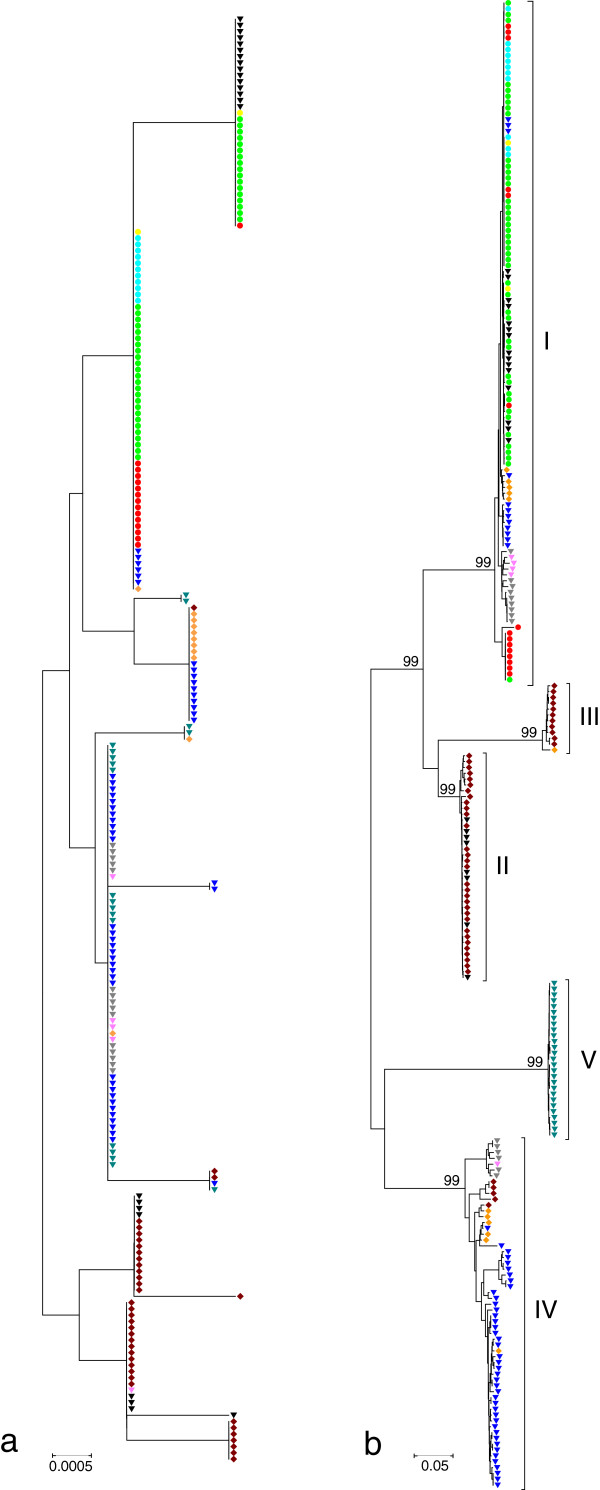
**Phylogenetic tree of the 16S rRNA (a) and *ankA *(b) gene sequences inferred using the neighbor-joining method.** Only bootstrap values exceeding 95% are shown. The scale bar indicates the number of nucleotide substitutions per site. **(a)** Sequences with ambiguous nucleotides were not included. The final data set contained 497 positions. **(b)** Only bootstrap values of major branches are shown. The final data set contained 2947 positions. Roman numerals indicate *ankA* gene clusters. Symbols: (light green circle) dog, (red circle) human, (light blue circle) horse, (yellow circle) cat, (inverted blue triangle) sheep, (inverted gray triangle) bison, (inverted pink triangle) cow, (orange diamond) red deer, (brown diamond) roe deer, (inverted blue green triangle) vole/shrew, (inverted black triangle) tick.

A neighbor-joining tree was constructed from the 34 *ankA* gene sequences obtained during this study and 221 sequences (12 from humans, 43 from dogs, 10 from horses, two from cats, 53 from sheep, four from cattle, 47 from roe deer, 12 from red deer, 15 from European bison, 23 from *I. ricinus* ticks) described earlier. As shown in Figure 1b, the *A. phagocytophilum* strains from voles and shrews were located on a distinct major branch that was supported by a high bootstrap value of 99%. As described previously [11], sequences from humans, dogs, horses, and cats were found exclusively in *ankA* gene cluster I. Sequences from sheep, cattle, red deer, and European bison were more heterogenous and belonged with the exception of one red deer sequence to *ankA* gene clusters I and IV. In contrast, sequences from roe deer were almost exclusively found in *ankA* gene clusters II and III. With the exception of *ankA* gene clusters III and V, sequences from *I. ricinus* ticks were scattered around the tree as expected. Using AnkA amino acid sequences similar results were obtained (data not shown). In contrast, on a tree calculated from the 16S rRNA gene sequences, no clear clustering was observed (Figure 1a).

### Recombination analysis

**Figure 2:**
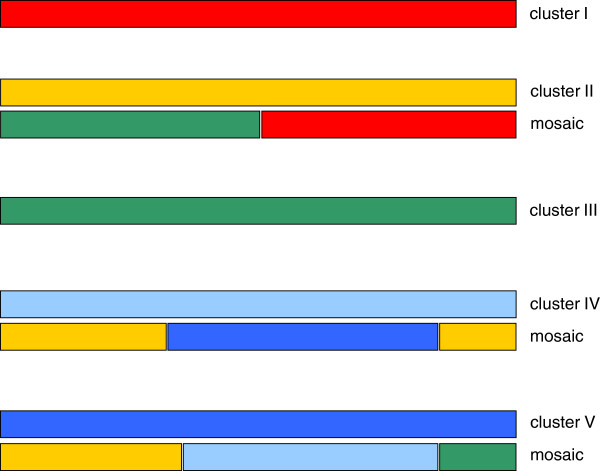
**Recombination analysis using the Recco method.** The *ankA* open reading frame of clusters I (red), II (yellow), III (green), IV (light blue), and V (dark blue) is shown. Underneath clusters II, IV, and V the conservative solutions from the analysis without repeats and gaps is demonstrated. Each solution is defined by the calculated recombination breakpoints and the sequence most similar to the putative recombinant between the breakpoints. The hypotheses were generated using the Recco method [41].

It is possible that the striking diversity of *ankA* gene sequences could have developed via recombination. To test this hypothesis, we generated nucleotide consensus sequences for each *ankA* gene cluster. A codon-based alignment of the five consensus sequences was created and analyzed applying the Recco method [41]. Because the sequences contained many repeats near their 3′ ends, the alignment was uncertain in the respective region and contained many gaps. Recco is subject to bias when analysing alignments with large gaps. We therefore further analyzed alignments without repeats as well as alignments without repeats and without any gaps. The results were compared to the analysis including repeats and all gaps. Whilst there was a tendency for Recco to report more and possibly spurious recombination events in the alignment containing repeats and gaps, we could confirm several recombination events with high confidence. Figure 2 shows the conservative solutions from the analysis without repeats and gaps. Each solution is defined by the calculated recombination breakpoints and the sequence most similar to the putative recombinant between the breakpoints.

## Discussion

In Europe, the reservoir hosts for *A. phagocytophilum* have not been clearly defined to date. The molecular characterization of *A. phagocytophilum* strains using *groESL* and *ankA* gene sequences revealed that red deer [11,30] and wild boar [31,32] might harbour variants that cause granulocytic anaplasmosis in humans, dogs, and horses. Small mammals were considered as reservoir hosts too, but it was shown that voles were infected with *msp4* genotypes that differed from those of *I. ricinus* ticks [34]. Because *I. ricinus* is the main vector of granulocytic anaplasmosis in humans and domestic animals in Europe [2], voles rather seem to be involved in a separate enzootic cycle probably with *I. trianguliceps* as tick vector [34]. This is in line with our observation that voles and shrews harboured *A. phagocytophilum* strains that belonged to a newly defined distinct *ankA* gene cluster. Interestingly, we did not find sequences from *I. ricinus* ticks to cluster with those from voles and shrews supporting the hypothesis that *A. phagocytophilum* strains circulating in these small rodents are part of a completely separate ecology [34]. Similarly, the *groESL* variants in voles and shrews from the Asian part of Russia were found to be clearly separated phylogenetically from all other analyzed strains [42]. This is in contrast to the USA, where small rodents such as the white-footed mouse appear to be reservoir hosts for granulocytic anaplasmosis [16,17]. Our results from the *ankA*-based phylogeny indicate that voles and shrews harbour *A. phagocytophilum* strains that might not be infectious for humans, dogs, horses, and livestock. However, other rodents species apart from those investigated here, could serve as reservoir hosts in Europe.

The AnkA protein is suggested to be secreted into host cells via the VirB/VirD-dependent type IV secretion system (T4SS) of *A. phagocytophilum*[45,46]. After translocation it is tyrosine phosphorylated and thought to disturb host cell signalling via protein-protein interactions mediated by its ankyrin repeats [45,46]. At its C-terminal end AnkA typically contains one classical EPIYA and multiple EPIYA-related motifs [11,47] that undergo tyrosine phosphorylation [47]. EPIYA motifs of bacterial effector proteins often show numerous duplications [44]. We described this phenomenon before especially for AnkA clusters I and IV [11] and show here that this is also true for the AnkA cluster V associated with voles and shrews (Additional file 3: Figure S1).

For the effector protein CagA of *Helicobacter pylori*, it was shown that its EPIYA motifs expanded via point mutation and recombination [48]. Our analysis of the five *ankA* consensus sequences revealed that the marked diversity of AnkA could have arisen via recombination as well (Figure 2). However, it was not possible to determine which sequences were the ancestral ones. It has been suggested that the diversification of EPIYA motifs may lead to altered or extended target-protein binding capacities [44]. Therefore, a specific AnkA could mediate a distinct host tropism of a particular *A. phagocytophilum* isolate and be involved in host adapation. Accordingly, variability between strains from different host species was found mainly in the surface-exposed components of the T4SS of *A. phagocytophilum*[49].

If the *ankA* gene is indeed involved in host adaptation driven by recombination, the *ankA*-based phylogeny could be disturbed by the fact that one single recombination event can introduce multiple nucleotide exchanges at once. Therefore, other more conserved loci should be used to proof the phylogenetic separation of *A. phagocytophilum* strains from voles and shrews described here. Nevertheless, their marked dissimilarity to all other strains investigated, indicates a long evolutionary distance. As sequence data alone are not able to prove different biological strain properties, in vivo experiments should address whether *A. phagocytophilum* isolates from voles and shrews are infectious for humans, dogs, horses, and livestock.

Although there might be some sampling error in our data set, voles and shrews are unlikely reservoir hosts for granulocytic anaplasmosis in humans, dogs, horses, and livestock in Europe based on *ankA* genotyping.

## Conclusions

Voles and shrews harbour *A. phagocytophilum* strains that contain *ankA* gene sequences belonging to the newly described cluster V that might have arisen via recombination. Because cluster V *ankA* sequences were restricted to voles and shrews, they are unlikely to serve as reservoir hosts for granulocytic anaplasmosis in humans, dogs, horses, and livestock in Europe.

## Consent

For Germany, permission to trap rodents using snap traps was given by the District Government Stuttgart, Germany [50]. For the United Kingdom, protocols for the handling and sampling of wild small mammals were approved by the University of Liverpool Committee on Research Ethics and were conducted in compliance with the terms and conditions of licenses awarded under the UK Government Animals (Scientific Procedures) Act, 1986 [34].

The samples of human and domestic animal origin were obtained as part of routine diagnostic evaluation. Informed consent was obtained from the patients and owners, respectively. Human samples 96 HE27 and 98 HE4 were kindly provided by Stephen J. Dumler (The Johns Hopkins School of Medicine, Baltimore, MD), human HGE-1 [36] and dog Martin [37] samples by Ulrike G. Munderloh (University of Minnesota, St. Paul, MN), horse sample 32 FR by Daniel Schaarschmidt-Kiener (Laboratory at Zugersee, Hünenberg, Switzerland) and cow A262 and sheep F1480 samples by Martin Ganter (University of Veterinary Medicine, Hannover, Germany).

## Availability of supporting data

All supporting data are included as additional files.

## Competing interests

The authors declare that they have no competing interests.

## Authors’ contributions

KH and RB did the sampling of the small mammal material and isolated the DNA. JM, NW, and FDvL performed DNA amplification and sequencing. FDvL carried out the data analysis and drafted the manuscript. The manuscript was critically read by JM, NW, KH and RB. All authors approved its final version.

## Supplementary Material

Additional file 1: Table S1 Primers used for amplification and sequencing of the complete ORF of *ankA* gene cluster V. Click here for file

Additional file 2: Table S2 Nucleotide sequences of the primers used for amplification and sequencing of the complete ORF of *ankA* gene cluster V. Click here for file

Additional file 3: Figure S1 Composition of the C-terminal end of cluster V AnkA. The composition of the C-terminal end of cluster V AnkA from 27 voles and shrews is shown. Homologous protein domains are displayed in same colors. Tyrosine phosphorylation motifs predicted by Scansite (http://scansite.mit.edu/) are indicated. Click here for file
