# Supplementary material for: Anaplasma phagocytophilum strains from voles and shrews exhibit specific ankA gene sequences
Source: BMC Vet Res. 2013 Nov 28;9:235. doi: 10.1186/1746-6148-9-235 (PMC4220824; doi:10.1186/1746-6148-9-235)
Supplement: Additional file 1: Table S1 — Primers used for amplification and sequencing of the complete ORF of ankA gene cluster V. [file 1746-6148-9-235-S1.doc]

**Additional file 1: Table S1.** Primers used for amplification and sequencing of the complete ORF of *ankA* gene cluster V

| **first PCR** | **nested PCR** | **sequencing** |
| --- | --- | --- |
| Nager U8  Nager beg re5 | Nager U8  Nager beg re6 | Nager beg re6, Nager U8 |
| Nager beg fo3  Nager beg re3 | Nager beg fo3  Nager beg re4 | Nager beg fo3, Nager beg re4 |
| Nager beg fo2a  Nager beg re1 | Nager beg fo2a  Nager beg re2 | Nager beg fo2a, Nager beg re2,  Nager beg seq1, Nager beg seq2 |
| Nager beg fo1a  Nager mi re8 | Nager beg fo1a  Nager mi re7 | Nager beg fo1a, Nager mi re7 |
| Nager mi fo6  Nager end re1 | Nager mi fo6  Nager end re4 | Nager end re4, Nager mi fo4, Nager mi fo6,  Nager mi re5, Nager mi re8, Nager mi seq2 |
| Nager end fo3  Nager D1 | Nager end fo4  Nager D1 | Nager end fo4, Nager D1 |
