# Supplementary material for: Anaplasma phagocytophilum strains from voles and shrews exhibit specific ankA gene sequences
Source: BMC Vet Res. 2013 Nov 28;9:235. doi: 10.1186/1746-6148-9-235 (PMC4220824; doi:10.1186/1746-6148-9-235)
Supplement: Additional file 2: Table S2 — Nucleotide sequences of the primers used for amplification and sequencing of the complete ORF of ankA gene cluster V. [file 1746-6148-9-235-S2.doc]

**Additional file 2: Table S2.** Nucleotide sequences of the primers used for amplification and sequencing of the complete ORF of *ankA* gene cluster V

| Primer name | Nucleotide sequence |
| --- | --- |
| Nager beg fo1a | GGG CTG GTC AAT ATG ATT TAG T |
| Nager beg fo2a | AGG TCT TTC AAAC GGC TCT CA |
| Nager beg fo3 | ATA CTG CCG TTG ATG CTA ATG |
| Nager beg re1 | ATA ACA GCT CTA GTC CTC TC |
| Nager beg re2 | ACT CCC TTA TTT GCG GCT TG |
| Nager beg re3 | CTT CAT CTA ACA CTG AGA ATG C |
| Nager beg re4 | CGC AAT GTG CCA TAT TCT TAG |
| Nager beg re5 | CAT CTA CAT CCT GAG CAC TT |
| Nager beg re6 | ATG TTT ACA TCG CAG TTC TGT |
| Nager beg seq1 | ATA TCT CTA AGT TTC CAT CCC |
| Nager beg seq2 | GCT GCA CGC CTG TTG TAA CT |
| Nager D1 | ATC TTA ACG ACT TTT CGG TGT AA |
| Nager end fo3 | GAG TAA GTC ATC TCC AGG TCA |
| Nager end fo4 | GGT GCT ACG GTA CTT ACT TC |
| Nager end re1 | GAT CCA TCT CCT GCC CTA CC |
| Nager end re4 | CTC TGA ATG ACT TGA AGC CTT |
| Nager mi fo4 | TAT GTT AGC AGG TTA TAG GAC TC |
| Nager mi fo6 | CTT AAG AAG CGT GTG TCA AAA G |
| Nager mi re5 | GCT CTG GAG TAG CAG TAC CTT |
| Nager mi re7 | ATA TCT GCA ACT GAT ACT TCT G |
| Nager mi re8 | TCA CTC TCC GAT TTA CTG CC |
| Nager mi seq2 | CAG ATA CAT CTC CTG GAA AAG |
| Nager U8 | ATT GAA TAA AGG CCC CAA CA |
