# Supplementary material for: Anaplasma phagocytophilum strains from voles and shrews exhibit specific ankA gene sequences
Source: BMC Vet Res. 2013 Nov 28;9:235. doi: 10.1186/1746-6148-9-235 (PMC4220824; doi:10.1186/1746-6148-9-235)
Supplement: Additional file 3: Figure S1 — Composition of the C-terminal end of cluster V AnkA. The composition of the C-terminal end of cluster V AnkA from 27 voles and shrews is shown. Homologous protein domains are displayed in same colors. Tyrosine phosphorylation motifs predicted by Scansite (http://scansite.mit.edu/) are indicated. [file 1746-6148-9-235-S3.ppt]

## Slide 1
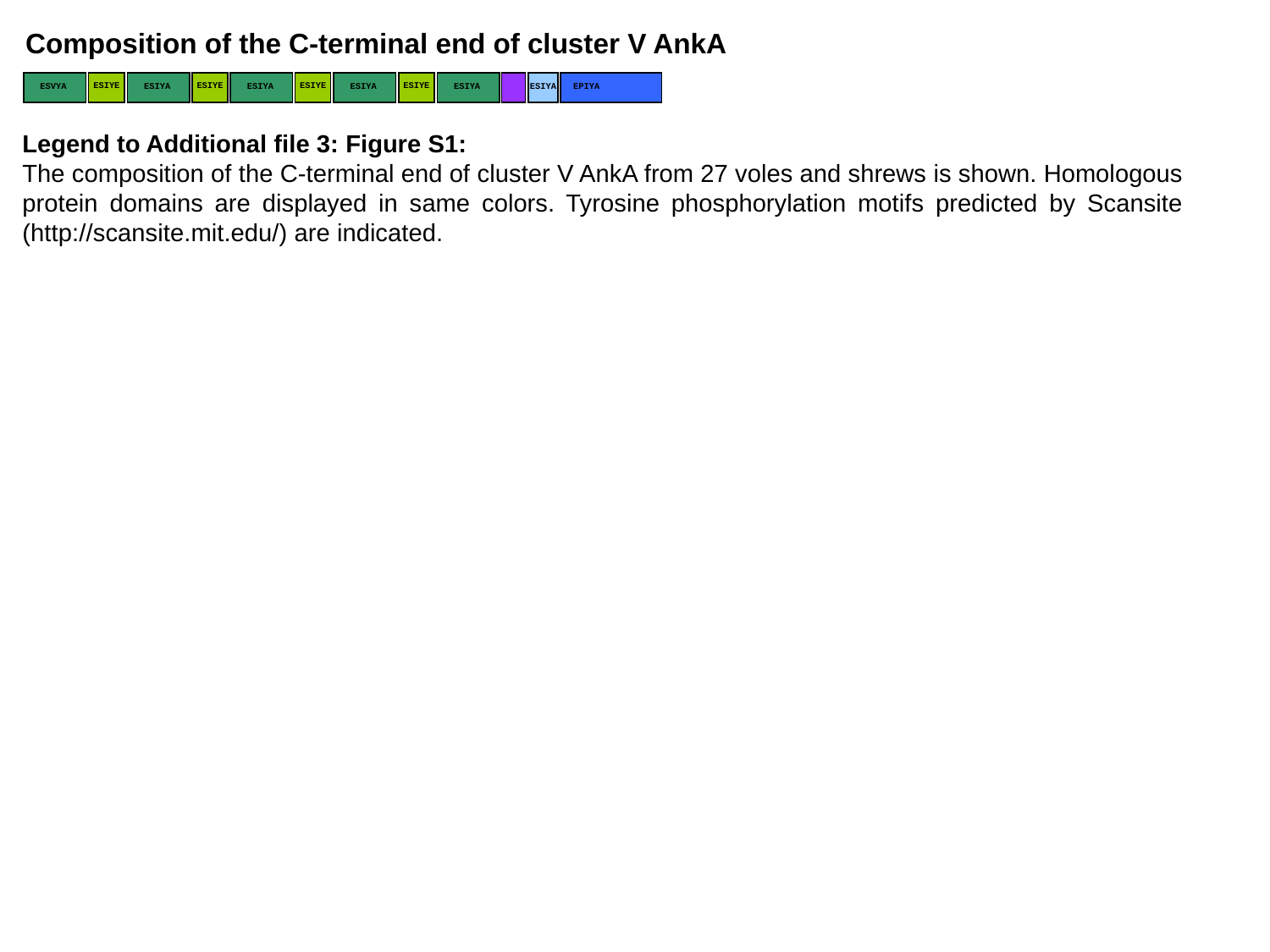

Composition of the C-terminal end of cluster V AnkA
ESVYA
ESIYE
ESIYA
ESIYE
ESIYA
ESIYE
ESIYA
ESIYE
ESIYA
ESIYA
EPIYA
Legend to Additional file 3: Figure S1:
The composition of the C-terminal end of cluster V AnkA from 27 voles and shrews is shown. Homologous protein domains are displayed in same colors. Tyrosine phosphorylation motifs predicted by Scansite (http://scansite.mit.edu/) are indicated.
